# Epidemiological Surveillance of Norovirus and Rotavirus in Sewage (2016–2017) in Valencia (Spain)

**DOI:** 10.3390/microorganisms8030458

**Published:** 2020-03-24

**Authors:** Cristina Santiso-Bellón, Walter Randazzo, Alba Pérez-Cataluña, Susana Vila-Vicent, Roberto Gozalbo-Rovira, Carlos Muñoz, Javier Buesa, Gloria Sanchez, Jesús Rodríguez Díaz

**Affiliations:** 1 Department of Microbiology, School of Medicine, University of Valencia, Av. Blasco Ibañez 17, 46010 Valencia, Spain; cristina.santiso@uv.es (C.S.-B.); walter.randazzo@uv.es (W.R.); susana.vila@uv.es (S.V.-V.); roberto.gozalbo@uv.es (R.G.-R.); carlos.munoz@uv.es (C.M.); javier.buesa@uv.es (J.B.); 2 Department of Preservation and Food Safety Technologies, IATA-CSIC, Av. Agustín Escardino 7, 46980 Paterna, Valencia, Spain; alba.perez@iata.csic.es (A.P.-C.); gloriasanchez@iata.csic.es (G.S.)

**Keywords:** rotavirus, norovirus, sewage, genotyping

## Abstract

The aim of the present study was to perform the molecular epidemiology of rotaviruses and noroviruses detected in sewage samples from a large wastewater facility from the city of Valencia, Spain. A total of 46 sewage samples were collected over a one-year period (September 2016 to September 2017). Norovirus and rotavirus were detected and quantified by RT-qPCR, genotyped by semi-nested RT-PCR and further characterized by sequencing and phylogenetic analyses. Noroviruses and rotaviruses were widely distributed in sewage samples (69.6% for norovirus GI, 76.0% norovirus GII, and 71.7% rotaviruses) and viral loads varied from 4.33 to 5.75 log PCRU/L for norovirus GI, 4.69 to 6.95 log PCRU/L for norovirus GII, and 4.08 to 6.92 log PCRU/L for rotavirus. Overall, 87.5% (28/32) of GI noroviruses could not be genotyped, 6.25% (2/32) of the samples contained GI.2 genotype, and another 6.25% (2/32) were positive for GI.4 genotype. The most common genotype of GII noroviruses was GII.2 (40%, 14/35), followed by GII.6 (8.6%, 3/35) and GII.17 (5.7%, 2/35) while the remaining GII strains could not be typed (45.7%, 16/35). Rotavirus VP4 genotype P[8] was the only one found in 19 out of 33 rotavirus-positive samples (57.7%). G2 was the most prevalent rotavirus VP7 genotype (15.2%, 5/33) followed by G3, G9, and G12, with two positive samples for each genotype (6.1%, 2/33). In one sample both G1 and G2 genotypes were detected simultaneously (3%). The results presented here show that the surveillance of noroviruses and rotaviruses in sewage is useful for the study of their transmission in the population and their molecular epidemiology.

## 1. Introduction

Diarrheal disease is the second cause of death in children under five years of age worldwide, producing around 525,000 deaths, with rotavirus and norovirus being responsible for the majority of the cases of non-bacterial acute gastroenteritis [1]. Norovirus is the leading cause of sporadic cases and outbreaks of acute gastroenteritis in children and adults [2,3], while rotavirus mainly causes disease in neonates and children under 5 years of age. Moreover, noroviruses show prolonged stability in environmental waters, resulting infectious in water for at least two months [4,5,6]. Worldwide, noroviruses are also the main cause of foodborne gastroenteritis outbreaks with an estimate of 120 million cases in 2010 [7]. The annual mortality associated with norovirus infections is of 200,000 deaths [8]. The genera *Norovirus* belongs to the *Caliciviridae* family and are further classified into ten distinct genogroups (GI–GX), that are subdivided into different genotypes [9]. Genogroups GI, GII, GIV, GVIII, and GXIX have been identified in infecting humans. Most of the human isolates belong to genogroups GI and GII that are further subdivided in 36 genotypes (GI.1-9, GII.1-28, and GII.15 has been withdrawn) [9]. The noroviruses are non-enveloped, icosahedral viruses with a single stranded, positive sense polyadenylated RNA genome [10]. The norovirus genome is organized into three open reading frames (ORFs), ORF2 encodes the major structural protein VP1 that forms the viral capsid with 180 copies structured in 90 dimers [11]. The VP1 protein can be divided in two regions, the shell (S) domain and the protruding (P) domain [12]. 

Rotavirus belongs to the *Reoviridae* family and are divided into at least eight different groups or species, called A to H [13]. Rotavirus from the groups A, B, and C infect humans, and many animal species. The rotavirus from group A is the most important group infecting humans and can be further classified into G (depending on VP7, which is a glycoprotein) and P (from the VP4 protein, that is sensitive to proteases) types. So far, at least 36 G-genotypes and 51 P-genotypes have been identified among human and animal rotaviruses [14]. Viruses carrying G1P[8], G2P[4], G3P[8], and G4P[8] represent over 90% of human rotavirus strains co-circulating in most countries, although other G and P combinations like G9P[8] and G12P[8] are being isolated in increasing numbers [15].

Due to the public health relevance of rotavirus and norovirus infections it is necessary to implement rapid molecular techniques to perform an epidemiological surveillance of these viruses. In fact, sewage surveillance is a powerful approach to study the epidemiology and distribution of human enteric viruses in a population. This is because this type of water receives faeces and vomit with norovirus and rotavirus from symptomatic and asymptomatic individuals, many of whom do not go to the hospital, making it more difficult to carry out a good epidemiological surveillance. Enteric virus detection and characterization in sewage has been broadly used worldwide to study the molecular epidemiology of rotaviruses and noroviruses in given population areas from the beginning of the present century, in both high income and low income countries [16,17,18,19,20,21]. The efforts have been mainly focused on detecting, quantifying, and characterizing gastroenteritis-producing viruses such as rotavirus, norovirus, and adenovirus, but also hepatotropic viruses such as hepatitis A and hepatitis E viruses [22,23]. Polioviruses and other enteroviruses have also been detected and characterized from sewage samples [18] as well as emergent viruses like Aichi virus [24]. Thus, environmental virology is a powerful tool to study relevant human viruses in given areas. Here we present the first study of molecular epidemiology of rotavirus and norovirus from sewage samples from a large wastewater facility in the city of Valencia from September 2016 to September 2017.

## 2. Materials and Methods

### 2.1. Sample Collection and Treatment

Samples were collected weekly from the Quart-Benager municipal wastewater treatment plant of Xirivella (Valencia) from September 2016 to September 2017. This treatment plant receives wastewater from 6 municipalities (western Valencia) with about 164,000 habitants and has an average flow of 30,318 m^3^/day.

Viral particles were concentrated by ultracentrifugation as previously done [18]. Briefly, 35 mL of sewage was centrifuged at 140,000× *g* for 2 h 30 min at 4 °C using a SW28 rotor. The elution of viral particles was achieved by adding 5 mL of 0.25 N glycine buffer (pH 9.5) to the sediment and incubating on ice for 30 min. The solution was neutralized by adding 5 mL of 2× phosphate-buffered saline. The suspended solids were removed by centrifugation (12,000× *g* for 15 min), and viruses were finally recovered by centrifugation at 229,600× *g* for 1 h at 4 °C in a 70Ti rotor. Viral particles were suspended in 500 µL of 1× PBS.

### 2.2. RNA Extraction and Molecular Detection of Noroviruses and Rotaviruses

Mengovirus (6 log PCRU/L, CECT 100000) was added to 35 mL of sewage as process control virus to monitor extraction efficiency following the ISO 15216:2017 guidelines. Viral RNA was extracted from 125 µL of sample concentrates with the NucleoSpin^®^ RNA Virus Kit (Macherey-Nagel, Düren, Germany), following the manufacturer’s instructions and including the Plant RNA Isolation Aid (Ambion, Austin, TX, USA) pre-treatment to remove potential PCR inhibitors such as polyphenolics and polysaccharides [25,26]. Then, RNA was finally eluted in 50 µL of RNase-free H_2_O. Mengovirus, rotavirus, norovirus GI, and GII amplification of direct and ten-folded diluted RNA samples was performed using one-step TaqMan RT-qPCR with the RNA UltraSense One-Step quantitative system (Invitrogen SA, Carlsbad, CA, USA) in the LightCycler 480 instrument (Roche Diagnostics, Risch-Rotkreuz, Switzerland). Each 10 µL reaction mix contained 2 µL of 2× master mix and 2.5 µL of RNA. Norovirus GI, GII, and mengovirus reaction mixes contained TaqMan^®^ probe at a final concentration of 250 nM, a reverse primer at 900 nM, and forward primer at 500 nM. For rotavirus, the TaqMan^®^ probe was used at a final concentration of 100 nM, and the primers were used at a final concentration of 250 nM (each). The set of primers and probes used for norovirus [27,28], rotavirus [29], and mengovirus [28,30] are reported in Table 1.

For mengovirus and norovirus GI and GII, RT-qPCR amplification was performed for 1 cycle at 55 °C for 1 h, 1 cycle at 95 °C for 5 min, and 45 cycles of 95 °C for 15 s, 60 °C for 1 min and 65 °C for 1 min [28]. For rotavirus, RT-qPCR amplifications were performed using the following conditions: Reverse transcriptase reaction for 30 min at 50 °C, followed by denaturation at 95 °C for 15 min, followed by 45 cycles of denaturation at 94 °C for 10 s, annealing at 55 °C for 30 s (fluorescence data collection at the end of annealing step), and extension at 72 °C for 20 s.

Standard curves for mengovirus, rotavirus, norovirus GI, and GII were generated by amplifying 10-fold dilutions of viral suspensions by RT-qPCR in quintuplicates.

Specifically, stool samples containing norovirus GI genotype 4 (GI.4) and GII genotype 4 (GII.4 variant Den Haag 2006b) were used to generate the standard curve. Stool samples were suspended (10%, wt/*v*) in phosphate-buffered saline (PBS) containing 2 M NaNO3 (Panreac, Barcelona, Spain), 1% beef extract (Condalab, Madrid, Spain), and 0.1% Triton X-100 (Fisher Scientific, Madrid, Spain) (pH 7.2), vortexed and centrifuged at 1000× *g* for 5 min. The supernatant was subsequently processed using the NucleoSpin^®^ RNA virus kit according to the manufacturer’s instructions.

Similarly, the standard curve for rotavirus was generated by serial end-point dilution, amplifying 10-fold dilutions of a quantified stock of Wa rotavirus strain (ATCC VR-2018) by RT-qPCR in quintuplicates. For each virus, the crossing points (Cp) obtained from the assay of each dilution were used to plot a standard curve by assigning a value of 1 RT-PCR unit (PCRU) to the highest dilution showing a positive Cp value and progressively 10-fold-higher values to the lower dilutions [31,32].

### 2.3. Sequencing and Phylogenetic Analysis

For the reverse transcription, 1 µL of 50 µM random primer (Biotools, Madrid, Spain) was added to 9 µL of each RNA sample denatured at 65 °C for 5 min. Each sample (RNA and random primer) contained a mixture of 1× Buffer (Invitrogen), 5 mM DTT (Invitrogen), 0.3 mM dNTPs (Biotools), 0.3 U RNasin (Biotools), and 0.2 µL of 1.3 U SuperScript^®^ III with a final reaction volume of 30 µL [33]. Then, each mixture was heated to 50 °C for 50 min for RT, followed by 70 °C for 15 min. Semi-nested RT-PCR assays were performed by standardized methods [33] in order to determine the G and P types of rotavirus. Briefly, the RNA extracted was reverse transcribed and amplified using consensus primer pairs VP7-F/VP7-R and VP4-F/VP4-R (Table 1), encoding the VP7 and VP4 genes as described previously. This was followed by semi-nested PCR using the specific primers that identify the most relevant G and P types [34] (Table 1). Genogroup I and II of norovirus were determined through semi-nested PCR using the primers COG1F-G1SKR and COG2F-G2SKR in a first round, respectively. The second round was done with the primers G1SKF-G1SKR and G2SKF-G2SKR, respectively [35,36] (Table 1).

The PCRs were performed as follow, 2.5 µL of cDNA were amplified in 1× Buffer with 2 mM MgCl_2_ (Biotools), 0.3 mM dNTPs (Biotools), 0.6 µM of each primer, and 1U DNA polymerase (Biotools) in a final volume of 25 µL. Then, each mixture was subjected to 94 °C for 2 min followed by 35 cycles of 94 °C for 30 s, 50 °C for 30 s, and 72 °C for 30 s. The final extension step was carried out at 72 °C for 10 min. PCR products were analyzed by gel electrophoresis on 2% agarose gels.

PCR products obtained from the second round of amplification were purified with GeneJET Gel Extraction or the GeneJET PCR Purification kit (Thermo Scientific) and sequenced in both directions. Sequencing was done by Sanger sequencing services from GATC Biotech. The quality of sequences was checked and manually corrected with BioEdit software v7.0.0 [37]. All sequences were deposited to GenBank accession number from MN621364 to MN621395 (rotavirus), MN602934 to MN602937 (norovirus GI), and from MN602951 to MN602969 (norovirus GII).

Phylogenetic trees were built with the sewage sequences and reference sequences obtained from Genbank. Sequences were aligned with the Clustal X 2.0 method [38] and matched with Genedoc 2.7.000 [39]. Finally, phylogenetic analyses were conducted using MEGA7 (Molecular Evolutionary Genetics Analysis v7.0 for bigger datasets) [40]. Models with the lowest BIC scores (Bayesian Information Criterion) are considered to describe the substitution pattern. In addition, the best model suggested by the program was used to calculate the degree of nucleotide sequence identity between the sequences studied. The evolutionary history was inferred by the Maximum Likelihood method [41] using a bootstrap test of 1000 replicates to assess tree reliability.

## 3. Results

### 3.1. Prevalence of Rotaviruses and Noroviruses in Sewage Samples from Valencia

In the present study a total of 46 sewage samples collected from September 2016 to September 2017 were analyzed. Table 2 summarizes the results including the data for norovirus GI, norovirus GII, and rotavirus. The recovery of spiked mengovirus was determined (Table 1) and ranged from 1.18% to 26.30%, complying with the recovery efficiency indicated in the ISO 15216-1:2017 to validate viral concentration in bottled water and food matrices (>1% of mengovirus recovery).

Regarding noroviruses, norovirus GII was the most prevalent genogroup as 76% of the samples (35/46) were positive, while norovirus GI was detected in 69.6% of the samples (32/46) by RT-qPCR. On average, the load (log PCRU/L) was slightly lower for norovirus GI (5.92 log PCRU/L) than for norovirus GII (6.05 log PCRU/L). Rotavirus was detected by RT-qPCR in 71.7% of the samples (33/46) with an average load of 6.92 log PCRU/L.

**Figure 1:**
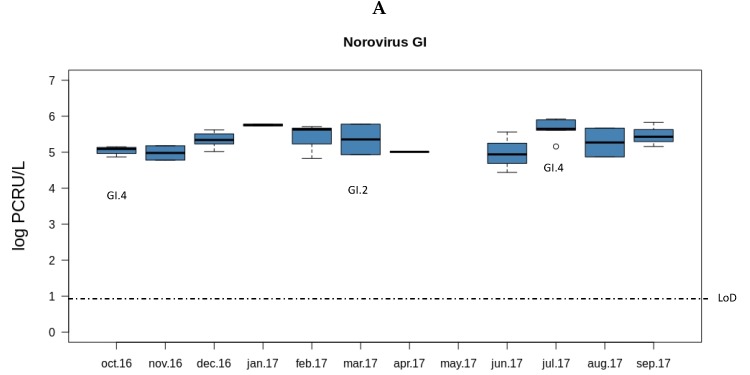

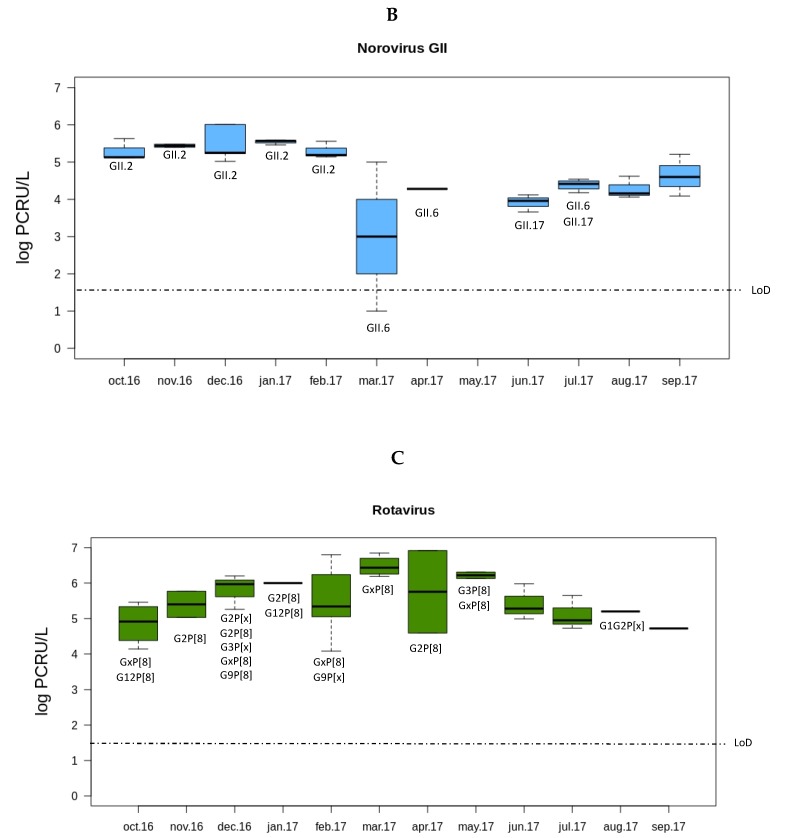
Overview of the concentrations and genotypes of enteric viruses detected in sewage. Boxplots show median concentrations (log PCRU/L) with the 25th and 75th percentile values of norovirus GI (**A** panel in dark blue), norovirus GII (**B** panel in light blue), and rotavirus (**C** panel in green). Viral genotypes are indicated according to the month in which the sample was sampled.

Both norovirus GII and rotavirus showed seasonality in the viral load along the year (Figure 1). The load of norovirus GII was higher during the cold months (October to February) and decreased sharply in spring (March). Interestingly, the months with lower norovirus GII loads were those with higher rotavirus loads (March to June). No seasonality was observed for norovirus GI.

### 3.2. Rotavirus and Norovirus Genotypes from Sewage Samples

In order to perform the genotyping of the samples, semi-nested RT-PCR was carried out as described in the Material and Methods section. Of the norovirus positive samples, 13 norovirus GI and 23 norovirus GII could be amplified. In a large number of samples, the genotype could not be determined (ND) even if the samples were positive by both RT-qPCR and semi-nested RT-PCR (Table 3). For the noroviruses GI, only four sequences were obtained out of the 13 seminested PCR positive samples (two sequences belonged to the GI.2 genotype and two to the GI.4 genotype). Within the GII genogroup, the most abundant genotype was the GII.2 (40%, 14/35). The second most common norovirus genotype was the GII.6 (8.6%, 3/35), followed by GII.17 (5.7%, 2/35).

Multiplex semi-nested RT-PCR was performed to study the genotypes of the rotavirus VP7 and VP4 coding genes (G and P genotypes respectively). In this case, 63% of the samples (29/46) could be amplified for at least one of these two targets. For VP7, the G2 genotype was the most prevalent (15.2%, 5/33). G3, G9, and G12 were detected with the same percentage, (6.1%, 2/33), followed by a sample with a mixture of genotypes G1+G2 (3%, 1/33). For the rotavirus VP4 gene, P[8] was the only genotype obtained in all the samples (57.7%, 19/33).

### 3.3. Phylogenetic Analyses of Sewage Detected Norovirus VP1 Coding Gene

**Figure 2:**
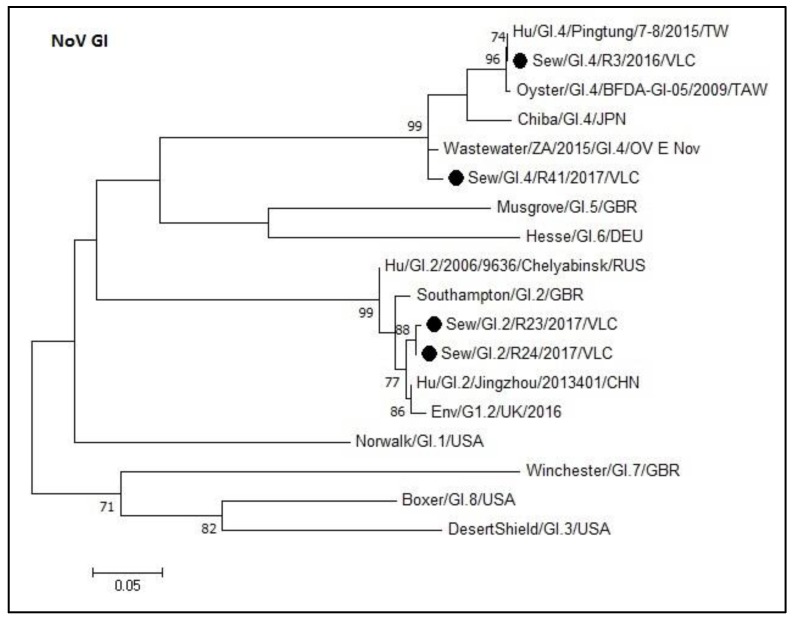
Molecular phylogenetic analysis of norovirus GI capsid. The evolutionary history was inferred using the Maximum Likelihood method based on the Kimura 2-parameter model [42] with a bootstrap of 1000 replicates. The tree is drawn to scale, the branch lengths measure the number of substitutions per site. The analysis included 18 nucleotide sequences. There were 276 positions in the final dataset including nucleotides from 5386 to 5662.

Figure 2 shows the phylogenetic tree of norovirus GI capsid sequences obtained in this study. Sequences formed monophyletic groups in the tree and are represented with their corresponding prototype strains within each genotype (GI.2 and GI.4). The degree of nucleotide sequence identity within the GI.2 sequences from this study ranged from 96.7% to 99.6%, while within the GI.4 sequences ranged from 91.3% to 100%. Furthermore, the percentage of nucleotide sequence identity between the GI.2 and GI.4 groups was 53.8% to 59.5%. Within the GI.4 group, the sequences from samples R3 and R41 shared an identity of 93.2%. R3 was identical to Pingtung sequence (MF996722.1). Regarding GI.2 group, the sequences from samples R23 and R24 shared an identity of 99.6%.

**Figure 3:**
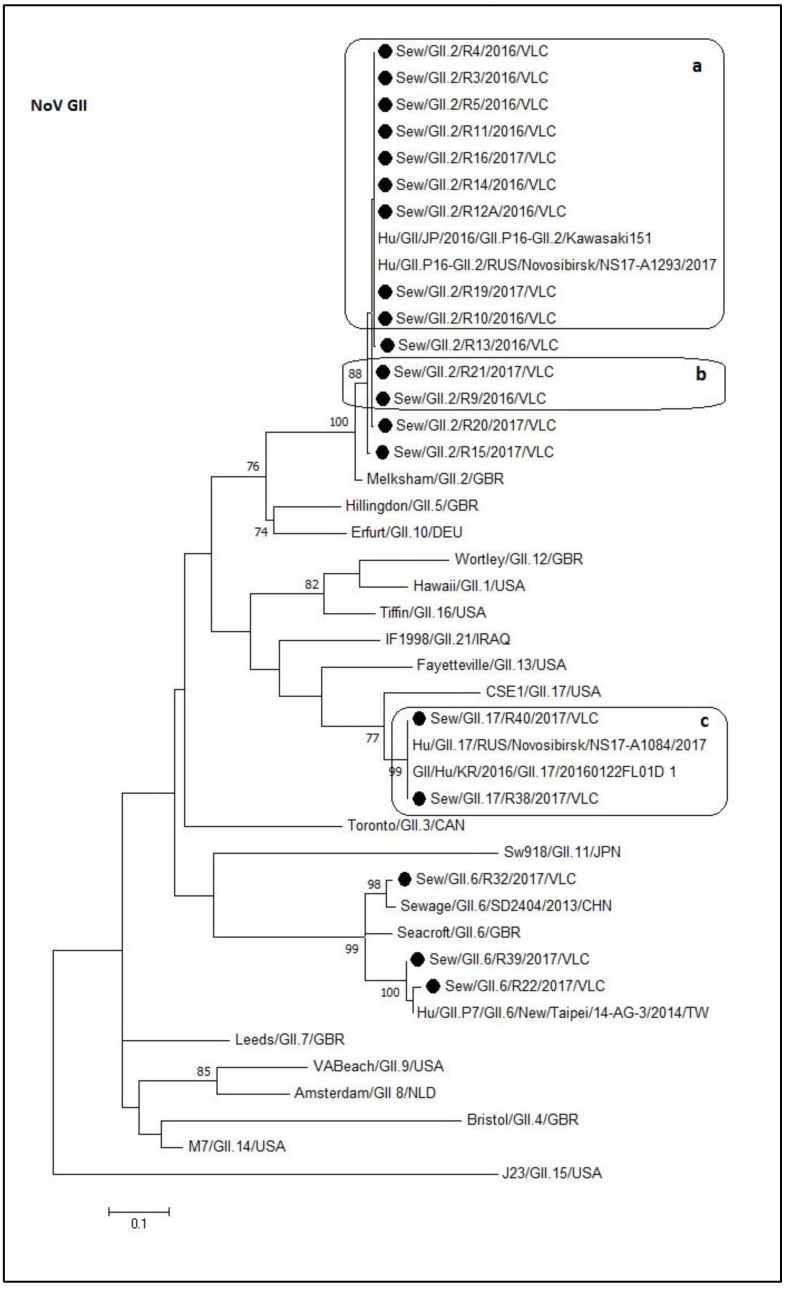
Molecular phylogenetic analysis of norovirus GII capsid. The evolutionary history was inferred by using the Maximum Likelihood method based on the Kimura 2-parameter model [42] with a bootstrap of 1000 replicates. The tree is drawn to scale, the branch lengths measure the number of substitutions per site. The analysis included 43 nucleotide sequences. There were 273 positions in the final dataset including nucleotides from 5113 to 5386. a, b, and c: Boxes that contain identical sequences.

The norovirus GII sequences from this study were divided in three well-supported clusters in the tree, corresponding to the GII.2, GII.6, and GII.17 genotypes (Figure 3). All the GII.2 sewage samples sequences grouped in the same cluster with a degree of identity between 95% and 100% compared to the reference strains. As observed in Figure 3, the different boxes include identical sequences (a, b, and c). All the sewage sequences included in the box ‘a’ were identical to two recombinant sequences of GII.P16-GII.2 genotype (Kawasaki151–LC215414.1, and Novosibirsk–MG893000.1). The GII.6 cluster grouped sequences with an identity between 88.1% and 98.9%. This cluster was divided into three subgroups. The first subgroup is composed of R32 and SD2404 (KR107699.1) sequences. The second include the SeaCroft reference sequence (AJ277620) while the last group contains R22, R39, and 14-AG-3 (KM036374.1) sequences. The identity within each subgroup was greater than 97.3%. Regarding the GII.17 cluster, it contains the box ‘c’ (100% of identity) sequences and a different subgroup composed of the reference sequence CSE1 (AY502009). This last sequence shared an identity of 81.3% with the box ‘c’ sequences.

### 3.4. Phylogenetic Analyses of Sewage Detected Rotavirus VP4 and VP7 Coding Genes

**Figure 4:**
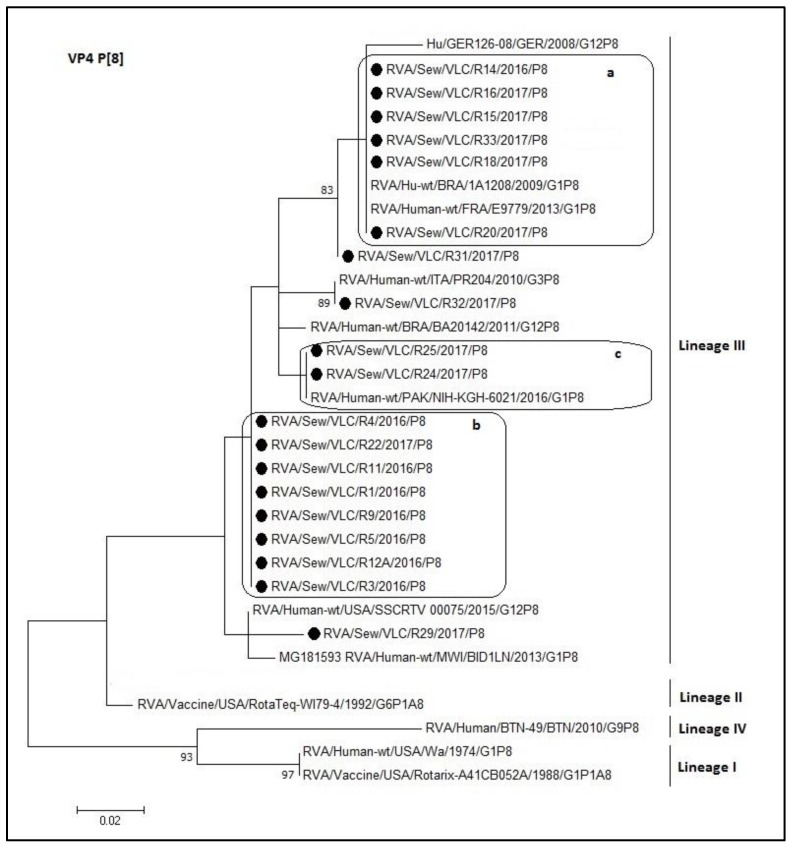
Molecular phylogenetic analysis of P[8] rotavirus (VP4 gene). The evolutionary history was inferred by using the Maximum Likelihood method based on the Tamura 3-parameter model [43] with a bootstrap of 1000 replicates. The tree is drawn to scale, the branch lengths measure the number of substitutions per site. The analysis included 32 nucleotide sequences. There were 127 positions in the final dataset. including nucleotides from 180 to 307. a, b, and c: Boxes containing identical sequences.

For the P[8] dendrogram of the rotavirus VP4 gene, all rotavirus sequences clustered within the lineage III (Figure 4). The nucleotide identity ranged from 92.7% to 100%. Sequences within a, b, and c boxes shared a 100% identity. Moreover, R32 is the same sequence like the PR204 Italian strain (KT988219.1). Nevertheless, the lower percentage of identity between sewage sample (lineage III) and reference sequences of different lineage was with lineage IV (73.1–82.8%). The nucleotide identity between the VP4 sequence of sewage samples and the vaccine strains was high, but not complete, RotaTeq^®^ shared an identity range of 90–95% and Rotarix^®^ shared an identity range of 84–90%.

**Figure 5:**
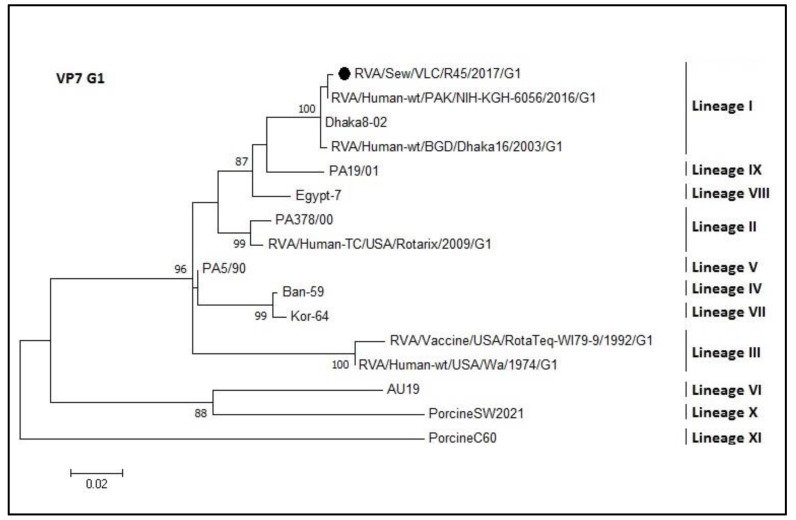
Molecular phylogenetic analysis of G1 rotavirus (VP7 gene). The evolutionary history was inferred by using the Maximum Likelihood method based on the Tamura 3-parameter model [43] with a bootstrap of 1000 replicates. The tree is drawn to scale, the branch lengths measure the number of substitutions per site. The analysis included 16 nucleotide sequences. There were 416 positions in the final dataset including nucleotides from 358 to 774.

**Figure 6:**
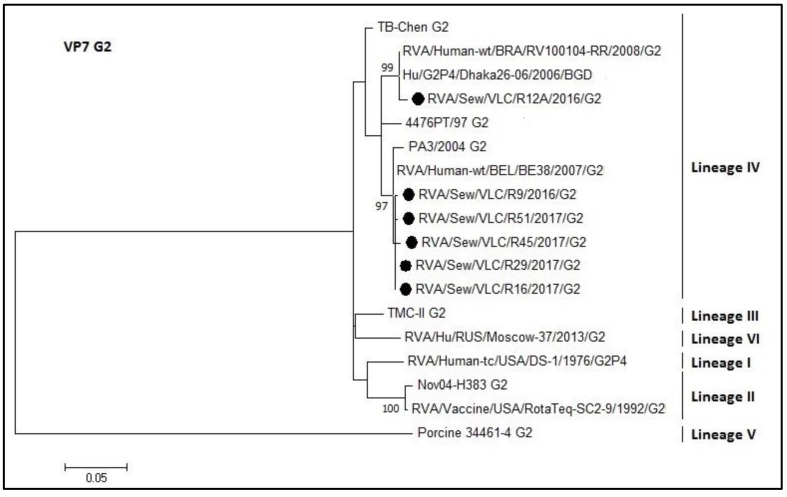
Molecular phylogenetic analysis of G2 rotavirus (VP7 gene). The evolutionary history was inferred by using the Maximum Likelihood method based on the Tamura 3-parameter model [43] with a bootstrap of 1000 replicates. The tree is drawn to scale, the branch lengths measure the number of substitutions per site. The analysis included 18 nucleotide sequences. There was a total of 460 positions in the final dataset including nucleotides from 424 to 884.

**Figure 7:**
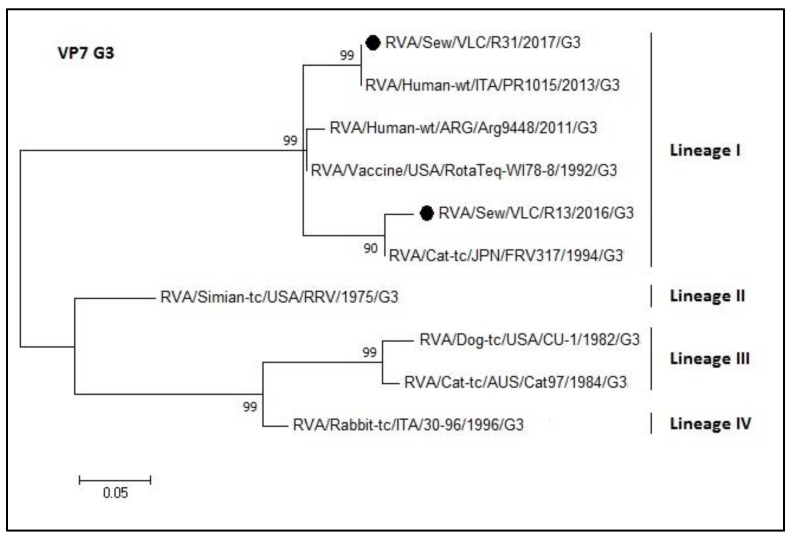
Molecular phylogenetic analysis of G3 rotavirus (VP7 gene). The evolutionary history was inferred by using the Maximum Likelihood method based on the Tamura 3-parameter model [43] with a bootstrap of 1000 replicates. The tree is drawn to scale, the branch lengths measure the number of substitutions per site. The analysis included 10 nucleotide sequences. There was a total of 159 positions in the final dataset including nucleotides from 774 to 933.

**Figure 8:**
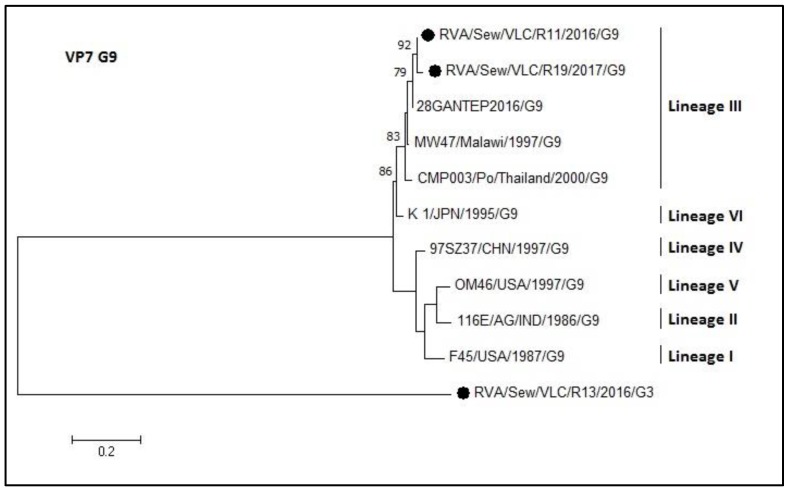
Molecular phylogenetic analysis of G9 rotavirus (VP7 gene). The evolutionary history was inferred by using the Maximum Likelihood method based on the Tamura 3-parameter model [43] with a bootstrap of 1000 replicates. The tree is drawn to scale, the branch lengths measure the number of substitutions per site. The analysis included 11 nucleotide sequences. There was a total of 159 positions in the final dataset including nucleotides from 774 to 933.

**Figure 9:**
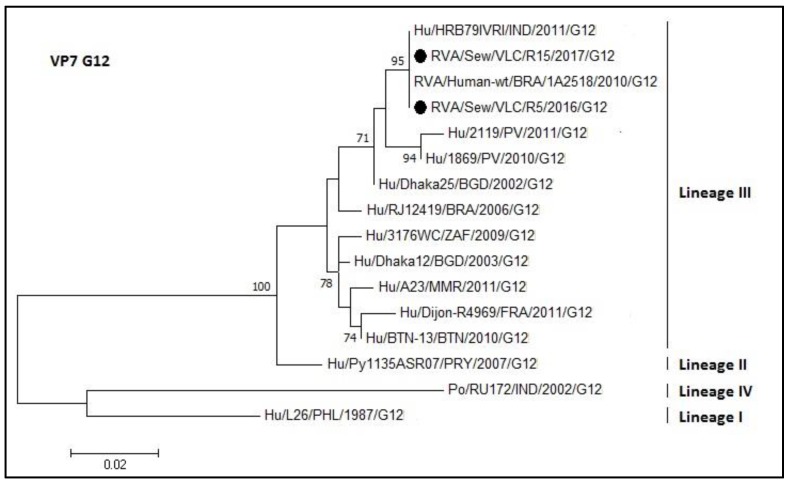
Molecular phylogenetic analysis of G12 rotavirus (VP7 gene). The evolutionary history was inferred by using the Maximum Likelihood method based on the Tamura 3-parameter model [43] with a bootstrap of 1000 replicates. The tree is drawn to scale, the branch lengths measure the number of substitutions per site. The analysis involved 16 nucleotide sequences. There was a total of 378 positions in the final dataset including nucleotides from 584 to 926.

Phylogenetic analysis of the VP7 coding gene of the rotavirus sequences was divided into five trees (each one for each of the sequenced genotypes G1, G2, G3, G9, and G12; Figure 5, Figure 6, Figure 7, Figure 8 and Figure 9, respectively). The R45 sample possessed a mixture of G1 and G2 genotypes. Figure 5 shows the tree of the G1 genotype. R45 grouped with sequences belonging to lineage I (nucleotide identity 99.3–99.8%). In this case, Rotarix^®^ was the vaccine strain with higher percentage of identity with the sewage sequence R45 (94%) while Rotateq^®^ shared an identity of 86%.

The rotavirus G2 genotype is shown in Figure 6. All sewage samples belonged to lineage IV (identity 96–100%). The R12A sequence grouped in a different cluster than the other sewage sequences. The percentage of identity between RotaTeq^®^ vaccine strain and the sewage samples ranged from 93% to 94%.

Figure 7 represents the phylogenetic tree of the G3 rotavirus genotype. The two sewage samples sequences (R13 and R31) clustered into lineage I (nucleotide identity 90–100%) although grouped into two different subgroups. The R31 sequence and the PR1015 (Italian sequence (KT988295.1)) shared a 100% nucleotide sequence identity. The R13 sequence grouped with a sequence belonging to a Japanese strain isolated from cat (FRV317, access number LC328208.1). On the other hand, RotaTeq^®^ G3 vaccine strain shared an identity of 93% and 96% with R13 and R31, respectively.

For the G9 dendrogram of the VP7 gene, the two sequences from sewage samples grouped in lineage III (Figure 8) sharing an identity of 94–98.7%. The R13 sample was typed as G9 by the multiplex PCR but by sequencing resulted to be an outgroup in that phylogenetic tree (G9 genotype) and was confirmed to be a G3 genotype (Figure 7).

The phylogenetic relationships of the G12 genotype are represented in Figure 9. The samples R5 and R15 have the same sequence and 100% nucleotide identity with a strain from India (HRB79; access number KC416951) and with a Brazilian strain (1A2518 access number KX932477.1). The sewage sample sequences belonged to lineage III.

## 4. Discussion

Sewage is an important source to study the epidemiology of viral pathogens transmitted by the faecal-oral route, especially when routine viral detection is not performed in outpatients or hospital facilities. The present study provided an overview of the epidemiology of noroviruses and rotaviruses through the analysis of sewage samples collected in Valencia from September 2016 to September 2017. In the present study, ultracentrifugation was selected as the concentration methodology since it was previously applied by our team successfully [18,22,24] and its performance showed to be comparable to other methods such as aluminium hydroxide adsorption–precipitation procedure [26]. Currently, RT-qPCR is the golden standard used to study viral loads [26,44,45] from sewage samples but next generation sequencing (NGS) methodologies have emerged strongly in the field in two modalities, sequencing the total virome with or without enrichment [46,47,48] and the NGS sequencing of PCR amplicons [49].

The present study has shown that both noroviruses and rotaviruses are widely disseminated (76% norovirus GII, 69.6% norovirus GI, and 71.7% rotaviruses) in the Valencian sewage and viral loads varied from 4.33 to 5.75 log PCRU/L, 4.69 to 6.95 log, and 4.08 to 6.92 log PCRU/L for norovirus GI, GII, and rotavirus, respectively.

For many of the positive samples obtained after conventional semi-nested RT-PCR, samples could not be genotyped due to mixed electropherograms. This fact may be due to the presence of more than one genotype in the same sample (a common trend in environmental samples). Despite this, the methodology applied allowed us to identify several noroviruses GI and GII. It is known that noroviruses GII have a wider circulation than noroviruses GI, playing a major role in acute gastroenteritis [15]. In the present study the capsid region that defines the norovirus genotype was targeted [9]. Most strains resulted to be of genogroup II, reflecting the higher circulation of the genogroup in the population [15].

In the last decades, the GII.4 noroviruses have been the most prevalent genotype infecting humans followed by others such as GII.2, GII.16, and GII.17 [9,50,51,52,53]. In the phylogeny of the norovirus GII (Figure 3) it can be observed that the GII.2 sequences grouped together with reference sequences of the recombinant genotype GII.P16-GII.2. Genetic recombination frequently occurs in RNA viruses and, since the first description of a naturally occurring norovirus recombination in 1997 [54], recombinant norovirus have been reported worldwide, including the region of Valencia [55]. Different studies have revealed an emergence of GII.P16-GII.2 recombinant strain in 2016 in different parts of the world [56,57,58,59]. This recombinant strain could become the currently predominant variant and be responsible for upcoming outbreaks of acute gastroenteritis caused by noroviruses. Monitoring of noroviruses in sewage is useful for the sensitive detection of epidemic variants in human populations.

It is known that rotaviruses causes disease in children younger than 5 years of age, especially in neonates and up to 2 years of age [60]. Therefore, most viruses remain in the diapers and do not travel into the sewage. This implies that the detected rotaviruses might be infecting older children and adults, probably asymptomatically. The large diversity of rotaviruses observed worldwide would theoretically allow up to 800 types of G- and P-type combinations, resulting in a wide variety of rotaviruses with different antigenic combinations [15]. In addition, it is possible to find mixed genotypes in which several G and/or P types are combined. This wide diversity can be generated by various mechanisms [61,62,63], which may lead to antigenic changes: Human-to-human or animal-to-human reassortments, interspecies transmission of rotavirus among multiple hosts, genetic drift, genetic recombination between different rotavirus strains, and inter- or intra-genic recombination. However, despite the wide antigenic and genomic variety of rotavirus, over the past three decades 90% of the strains identified in humans worldwide have presented the G1P[8], G2P[4], G3P[8], G4P[8], G9P[8], and G12P[8] genotypes, with different variations in geographic and temporal distribution [62,64,65,66,67]. The sewage rotavirus strains characterized in the present study belong to the P[8] lineage III, a lineage different from the vaccine strains Rotarix (P[8] lineage I) and RotaTeq- WI79-4 (P[8] lineage II). In addition, the P[8]-III strains segregated into distinct sub clusters (Figure 4). P[8] is currently the most common genotype infecting humans [64]. On the other hand, we can observe that sequences of the group ‘a’ (Figure 4 box ‘a’) share 100% identity with strains that have been circulating prior to this study (with the Brazilian strain 1A1208, 2009; and with the French strain E9779, 2013).

Interestingly, the R29 sample presented the G2P[8] combination. The recombination between G2 and P[8] is considered unusual. Figure 4 shows that the VP8* sequence of the R29 sample groups in a different cluster than the rest of the P[8] sequences (lineage III). Furthermore, the similarity between R29 and the rest of the sequences of lineage III presented a range between 92.7% (GER126-08) and 98.3% (SSCRTV_00075), the latter grouped in the same cluster. These identity percentages are lower than expected for sequences from a nearby common ancestor, where the most likely evolutionary mechanism is due to genetic drift. Interestingly, R29 obtained a higher identity with the RotaTeq vaccine strain (93.5%), belonging to lineage II, than with the GER1236-08 strain, belonging to lineage III. These data suggest a different mechanism of evolution and different origin of R29 strain compared to the rest of strains of lineage III. Thus, R29 cluster could constitute a sub-lineage of the lineage III, even a new lineage.

When the strains of this study were compared with vaccine strains the similarity of the deduced amino acid sequences was higher than nucleotide sequences. Regarding P[8] sequences, vaccines strains shared a similarity range of 95.1–97.6% with the strains analyzed. Within G1 genotype, the sequences of the sewage samples shared between 95.6% and 97.8% similarity with RotaTeq and Rotarix strains, respectively. In regard to G2 genotype, the similarity was 96.2–98.1%. Finally, the G3 genotype analyses of similarity showed a 95.6% identity between the RotaTeq strain and the sewage strains. These data suggest a high preservation of the function and structure of antigenic epitopes between these strains.

The relationship between the genotypes of rotavirus and norovirus detected in sewage water and the clinical isolates are unclear, since there are not published data on the epidemiology of clinical samples in this region for the studied period. However, we recently published the rotavirus clinical data for the period 2013–2015 [68] where the P[8] was the predominant genotype responsible of 97.7% of the clinical cases caused by rotavirus and was combined with G9 (49.6%), G1 (20.3%), and G12 (14.3%) VP7 genotypes. Interestingly, during 2013–2015 only 1.5 % of the samples where G2 and this is the genotype with a higher prevalence in sewage in the period 2016–2017. This might be due to a shift in the circulating genotypes in the population, to a higher stability of the G2 genotype in environmental samples or to the existence of a higher proportion of asymptomatic infections of rotaviruses of the G2 genotype compared to the G1, G9, and G12 genotypes.

As a conclusion, the results presented here show that the monitoring of noroviruses and rotaviruses in sewage is useful to study the molecular epidemiology of those viruses in a given population.

## Figures and Tables

**Table 1:** Sequences of primers and probes utilized in the present study. The asterisk (*) indicate the names of the primers used for sequencing.

Target	Purpose	Primer or Probe Name	Sequence	Nucleotide Positions
Norovirus GI	qPCR	QNIF4	5′-CGC TGG ATG CGN TTC CAT-3′	5291–5308
Norovirus GI	qPCR	NV1LCR	5′-CCT TAG ACG CCA TCA TCA TTT AC-3′	5354–5376
Norovirus GI	qPCR	NVGG1p	VIC-5′-TGG ACA GGA GAY CGC RAT CT-3′-Quencher	5321–5340
Norovirus GII	qPCR	QNIF2	5′-ATG TTC AGR TGG ATG AGR TTC TCW GA-3′	5012–5037
Norovirus GII	qPCR	COG2R	5′-TCG ACG CCA TCT TCA TTC ACA-3′	5080–5100
Norovirus GII	qPCR	QNIFs	FAM-5′-AGC ACG TGG GAG GGC GAT CG-3′-Quencher	5042–5061
Mengovirus	qPCR	Mengo 110	5′-GCG GGT CCT GCC GAA AGT-3′	110–127
Mengovirus	qPCR	Mengo 209	5′-GAA GTA ACA TAT AGA CAG ACG CAC AC-3′	245–270
Mengovirus	qPCR	Mengo 147	FAM-5′-ATC ACA TTA CTG GCC GAA GC-3′-Quencher	209–227
Norovirus GI	1st PCR	COG1F	5′-CGY TGG ATG CGN TTY CAT GA-3′	5291–5311
Norovirus GI	1st PCR/2nd PCR	* G1SKR	5′-CCA ACC CAR CCA TTR TAC A-3′	5653–5671
Norovirus GI	2nd PCR	* G1SKF	5′-CTG CCC GAA TTY GTA AAT GA-3′	5342–5361
Norovirus GII	1st PCR	COG2F	5′-CAR GAR BCN ATG TTY AGR TGG ATG AG-3′	5003–5029
Norovirus GII	1st PCR/2nd PCR	* G2SKR	5′-CCR CCN GCA TRH CCR TTR TAC AT-3′	5379–5401
Norovirus GII	2nd PCR	* G2SKF	5′-CNT GGG AGG GCG ATC GCA A-3′	5058–5076
Rotavirus NSP3	qPCR	JVKF	5′-CAG TGG TTG ATG CTC AAG ATG GA-3′	17–39
Rotavirus NSP3	qPCR	JVKR	5′-TCA TTG TAA TCA TAT TGA ATA CCC A-3′	123–147
Rotavirus NSP3	qPCR	JVKP	FAM-5′-ACA ACT GCA GCT TCA AAA GAA GWG T-3-Quencher	72–96
Rotavirus VP7	1st PCR	VP7-F	5′-ATG TAT GGT ATT GAA TAT ACC AC-3′	51–71
Rotavirus VP7	1st PCR/2nd PCR	* VP7-R	5′-AAC TTG CCA CCA TTT TTT CC-3′	914–932
Rotavirus VP7	2nd PCR	* G1	5′-CAA GTA CTC AAA TCA ATG ATG G-3′	314–335
Rotavirus VP7	2nd PCR	* G2	5′-CAA TGA TAT TAA CAC ATT TTC TGT G-3′	411–435
Rotavirus VP7	2nd PCR	* G3	5′-ACG AAC TCA ACA CGA GAG G-3′	250–269
Rotavirus VP7	2nd PCR	G4	5′-CGT TTC TGG TGA GGA GTT G-3′	480–499
Rotavirus VP7	2nd PCR	G8	5′-GTC ACA CCA TTT GTA AAT TCG-3′	178–198
Rotavirus VP7	2nd PCR	* G9	5′-CTT GAT GTG ACT AYA AAT AC-3′	757–776
Rotavirus VP7	2nd PCR	G10	5′-ATG TCA GAC TAC ARA TAC TGG-3′	666–687
Rotavirus VP7	2nd PCR	* G12	5′-GGT TAT GTA ATC CGA TGG ACG-3′	548–567
Rotavirus VP4	1st PCR/2nd PCR	* VP4-F	5′-TAT GCT CCA GTN AAT TGG-3′	132–149
Rotavirus VP4	1st PCR	VP4-R	5′-ATT GCA TTT CTT TCC ATA ATG-3′	775–795
Rotavirus VP4	2nd PCR	P[4]-R	5′-CTA TTG TTA GAG GTT AGA GTC-3′	474–494
Rotavirus VP4	2nd PCR	P[6]-R	5′-TGT TGA TTA GTT GGA TTC AA-3′	259–278
Rotavirus VP4	2nd PCR	* P[8]-R	5′-TCT ACT GGR TTR CAN TGC-3′	339–356
Rotavirus VP4	2nd PCR	P[9]-R	5′-TGA GAC ATG CAA TTG GAC-3′	385–402
Rotavirus VP4	2nd PCR	P[10]-R	5′-ATC ATA GTT AGT AGT CGG-3′	575–594
Rotavirus VP4	2nd PCR	P[11]-R	5′-GTA AAC ATC CAG AAT GTG-3′	305–323

**Table 2:** Prevalence, viral loads (log_10_ PCRU/L) and genotypes of norovirus GI, norovirus GII, and rotavirus.

Sample	Sample	Sampling Date	Norovirus				Rotavirus		Recovery Efficiency
			GI Norovirus		GII Norovirus				
			Genotype	log PCRU/L ± SD	Genotype	log PCRU/L ± SD	Genotype	log PCRU/L ± SD	%
R1	#1	29.9.16	ND	4.98 ± 0.43	ND	6.13 ± 0.05	GxP[8]	5.21 ± 0.08	1.47
R3	#2	18.10.16	GI.4	4.74 ± 0.32	GII.2	6.12 ± 0.35	GxP[8]	5.46 ± 0.14	5.11
R4	#3	20.10.16	ND	5.01 ± 0.25	GII.2	6.60 ± 0.01	GxP[8]	4.62 ± 0.18	2.22
R5	#4	27.10.16	ND	4.92 ± 0.06	GII.2	6.13 ± 0.12	G12P[8]	4.14 ± 0.10	4.37
R9	#5	17.11.2016	ND	4.66 ± 0.64	GII.2	6.39 ± 0.00	G2P[8]	5.03 ± 0.06	7.73
R10	#6	24.11.2016	ND	5.04 ± 0.28	GII.2	6.46 ± 0.11	ND	5.77 ± 0.22	6.39
R11	#7	2.12.2016	ND	4.89 ± 0.06	GII.2	6.24 ± 0.15	G9P[8]	5.26	1.37
R12A	#8	6.12.2016	ND	5.47 ± 0.43	GII.2	6.95 ± 0.10	G2P[8]	<LOD	7.62
R13	#9	21.12.2016	ND	5.09 ± 0.13	GII.2	6.02 ± 0.00	G3P[x]	5.97	6.73
R14	#10	30.12.2016	ND	5.36 ± 0.19	GII.2	6.95 ± 0.05	GxP[8]	6.20 ± 0.12	7.38
R51	#11	15.12.2016	ND	5.19 ± 0.32	ND	6.22 ± 0.13	G2P[x]	<LOD	8.54
R15	#12	4.01.2017	ND	5.62 ± 0.19	GII.2	6.44 ± 0.10	G12P[8]	<LOQ	7.73
R16	#13	11.01.2017	ND	5.57 ± 0.08	GII.2	6.56 ± 0.06	G2P[8]	6.00 ± 0.10	7.44
R17	#14	18.01.2017	ND	<LOD	ND	6.54 ± 0.18	ND	<LOD	8.38
R18	#15	15.02.2017	ND	<LOD	ND	<LOD	GxP[8]	6.24 ± 0.05	3.68
R19	#16	16.02.2017	ND	4.70 ± 0.21	GII.2	6.14 ± 0.09	G9P[x]	5.34 ± 0.04	4.79
R20	#17	2.02.2017	ND	5.55 ± 0.04	GII.2	6.53 ± 0.34	GxP[8]	5.05 ± 0.09	10.82
R21	#18	22.02.2017	ND	5.47 ± 0.08	GII.2	6.19 ± 0.76	ND	4.08 ± 0.23	4.39
R22	#19	02.03.2017	ND	<LOD	GII.6	5.38 ± 0.21	GxP[8]	6.32 ± 0.81	18.39
R23	#20	08.03.2017	GI.2	<LOD	ND	6.25	ND	<LOD	20.50
R24	#21	15.03.2017	GI.2	5.62 ± 0.05	ND	5.01 ± 1.71	GxP[8]	6.55 ± 0.25	12.50
R25	#22	23.03.2017	ND	4.80 ± 0.02	ND	5.82 ± 0.05	GxP[8]	6.85 ± 0.22	11.39
R26	#23	30.03.2017	ND	<LOD	ND	<LOD	ND	6.19 ± 0.08	1.18
R27	#24	06.04.2017	ND	<LOD	ND	<LOD	ND	<LOD	1.97
R28	#25	12.04.2017	ND	4.87 ± 0.10	ND	5.34 ± 0.00	ND	6.92 ± 0.04	2.82
R29	#26	27.04.2017	ND	<LOD	ND	<LOD	G2P[8]	4.59	4.31
R30	#27	04.05.2017	ND	<LOD	ND	<LOD	ND	6.31 ± 0.04	4.75
R31	#28	11.05.2017	ND	<LOD	ND	<LOD	G3P[8]	<LOD	11.03
R32	#29	18.05.2017	ND	<LOD	GII.6	<LOD	GxP[8]	6.13 ± 0.01	9.05
R33	#30	25.02.2017	ND	<LOD	ND	<LOD	GxP[8]	6.80	9.34
R34	#31	01.06.2017	ND	<LOD	ND	5.17	ND	<LOD	6.73
R35	#32	08.06.2017	ND	<LOD	ND	<LOD	ND	4.99	2.02
R36	#33	15.06.2017	ND	4.33 ± 0.45	ND	<LOD	ND	5.28	7.10
R37	#34	22.06.2017	ND	4.81 ± 0.57	ND	4.69 ± 0.00	ND	<LOD	9.40
R38	#35	28.06.2017	ND	5.41 ± 0.01	GII.17	5.04 ± 0.27	ND	5.98	2.48
R39	#36	05.07.2017	ND	5.45 ± 0.26	GII.6	5.43 ± 1.58	ND	5.65	1.53
R40	#37	06.07.2017	ND	5.03 ± 0.02	GII.17	<LOD	ND	4.95	3.53
R41	#38	13.07.2017	GI.4	5.73 ± 0.54	ND	5.50 ± 0.15	ND	<LOD	8.04
R42	#39	20.07.2017	ND	5.75 ± 0.31	ND	5.25 ± 0.00	ND	4.73 ± 0.10	2.34
R43	#40	27.07.2017	ND	5.49 ± 0.22	ND	5.58 ± 0.13	ND	<LOD	26.30
R44	#41	10.08.2017	ND	4.75 ± 0.37	ND	5.23 ± 0.37	ND	<LOD	13.44
R45	#42	17.08.2017	ND	5.51 ± 0.00	ND	5.65 ± 0.11	G1G2P[x]	<LOD	25.72
R46	#43	30.08.2017	ND	<LOD	ND	5.13 ± 0.18	ND	5.20 ± 0.07	10.05
R48	#44	7.09.2017	ND	5.02 ± 0.17	ND	5.16 ± 0.00	ND	<LOD	9.86
R49	#45	13.09.2017	ND	5.67 ± 0.06	ND	5.64 ± 0.09	ND	<LOD	4.24
R50	#46	20.09.2017	ND	5.28 ± 0.08	ND	6.19 ± 0.09	ND	4.72 ± 0.13	13.27

**Table 3:** Prevalence of norovirus GI, norovirus GII, and rotavirus in sewage samples from Valencia. ND is indicated when the genotype was not determined.

Norovirus			Rotavirus		
Genotype	GI (%)	GII (%)	Genotype	VP7 (%)	VP4 (%)
ND	87.5 (28/32)	45.7 (16/35)	ND	63.5 (21/33)	42.3 (14/33)
GI.2	6.25 (2/32)	-	P[8]	-	57.7 (19/33)
GI.4	6.25 (2/32)	-	G1+G2	3 (1/33)	-
GII.2	-	40 (14/35)	G2	15.2 (5/33)	-
GII.6	-	8.6 (3/35)	G3	6.1 (2/33)	-
GII.17	-	5.7 (2/35)	G9	6.1 (2/33)	-
			G12	6.1 (2/33)	-
